# Examining the Evidence for an Adult Healthy Middle Ear Microbiome

**DOI:** 10.1128/mSphere.00456-19

**Published:** 2019-09-04

**Authors:** Jake Jervis-Bardy, Lex E. X. Leong, Lito E. Papanicolas, Kerry L. Ivey, Sharad Chawla, Charmaine M. Woods, Claire Frauenfelder, Eng H. Ooi, Geraint B. Rogers

**Affiliations:** a Department of Otolaryngology—Head & Neck Surgery, Flinders Medical Centre, College of Medicine and Public Health, Flinders University, Adelaide, South Australia, Australia; b Department of Otolaryngology—Head & Neck Surgery, The University of Adelaide, Adelaide, South Australia, Australia; c Infection and Immunity Theme, South Australian Health and Medical Research Institute, Adelaide, South Australia, Australia; d SAHMRI Microbiome Research Laboratory, Flinders University School of Medicine, Adelaide, South Australia, Australia; e Department of Nutrition, Harvard T.H. Chan School of Public Health, Boston, Massachusetts, USA; The Jackson Laboratory for Genomic Medicine

**Keywords:** microbiome, middle ear, otitis media

## Abstract

Otitis media (OM) is a cluster of diseases of the middle ear that commonly result from bacterial infection. OM subtypes in which the tympanic membrane is intact (acute otitis media and otitis media with effusion) are presumed to result from pathogen translocation through the eustachian tube. Recent molecular-based studies have suggested that a diverse middle ear microbiome exists in the absence of disease. These have been largely unsupported by culture and feature species that commonly contaminate low-biomass sequencing data. Combining culture-based and molecular techniques, we undertook a detailed investigation of the evidence for bacterial colonization of the healthy middle ear. Middle ear (ME), nasopharynx (NP), and external ear canal (EC) swabs were collected from a total of 25 adult patients undergoing cochlear implant, stapedotomy, or translabyrinthine vestibular schwannoma resection. Diagnostic culture, microscopy, quantitative PCR, and 16S rRNA gene amplicon sequencing were used to assess sample bacterial content. EC and NP microbiota were consistent with previous reports. In contrast, bacterial levels in ME samples were not significantly above those in unused control swabs. Commonly detected taxa were among recognized sequencing contaminants (*Methylobacterium*, *Pseudomonas*, and *Acinetobacter*). Linear regression of dominant ME taxa confirmed a negative relationship between relative abundance and bacterial load, consistent with contamination. No bacteria were detected by microscopy or diagnostic culture in any middle ear sample. Our findings cast substantial doubt on previous reports identifying a healthy middle ear microbiome using 16S amplicon sequencing.

**IMPORTANCE** Recent molecular-based studies have suggested that a diverse middle ear microbiome in adults and children can exist in the absence of disease. These studies have been largely unsupported by culture and feature species that commonly contaminate low-biomass sequencing data. While 16S rRNA gene amplicon sequencing has proven to be a highly informative technique in many clinical contexts, it is susceptible to spurious signal arising from sequencing reagent contaminants where sample biomass is low. Combining culture-based and molecular techniques, we undertook a detailed investigation of the evidence for bacterial colonization of the healthy middle ear. In finding no evidence of viable bacterial cells in middle ear samples, our study further underlines the importance of careful consideration of amplicon sequence data derived from very-low-biomass contexts and the value of analytical approaches that combine culture and molecular techniques.

## Abstract (precis)

Recent molecular-based studies have suggested that a diverse middle ear microbiome in adults and children can exist in the absence of disease. These studies have been largely unsupported by culture and feature species that commonly contaminate low-biomass sequencing data. While 16S rRNA gene amplicon sequencing has proven to be a highly informative technique in many clinical contexts, it is susceptible to spurious signal arising from sequencing reagent contaminants where sample biomass is low. Combining culture-based and molecular techniques, we undertook a detailed investigation of the evidence for bacterial colonization of the healthy middle ear. In finding no evidence of viable bacterial cells in middle ear samples, our study further underlines the importance of careful consideration of amplicon sequence data derived from very-low-biomass contexts and the value of analytical approaches that combine culture and molecular techniques.

## INTRODUCTION

Otitis media (OM) represents a cluster of diseases of the middle ear, which includes acute otitis media (AOM) and otitis media with effusion (OME). The contribution of bacterial infection to AOM and OME has been the subject of widespread investigation. Culture-based studies of middle ear fluid (MEF) collected by needle aspiration or after myringotomy have reported Streptococcus pneumoniae, Haemophilus influenzae, Moraxella catarrhalis, and Streptococcus pyogenes to be commonly present (1–3).

Given that the tympanic membrane is intact in AOM and OME, the presence of microorganisms in the middle ear raises the question of their origin. It is widely presumed that infections result from bacterial translocation from the nasopharynx via the eustachian tube (4, 5). More recently, it has been proposed that ear canal bacteria also contribute to OM pathogenesis, entering the middle ear during perforation-healing cycles in individuals that have recurrent episodes of acute otitis media with resulting perforation (6). However, whether bacteria colonize the middle ear mucosa in the absence of inflammation or current disease is more contentious.

A number of attempts have been made to detect bacteria within the middle ear in the absence of infection, employing a range of analytical strategies. The first two studies that attempted to address this question used scanning electron microscopy to visualize microbes from middle ear mucosa obtained during cochlear implantation (7, 8). Of these, only one was successful in detecting bacterial cells (7), and the species to which they belonged was not determined. A subsequent PCR-based microbial identification study failed to detect any bacterial signal in 22 healthy middle ears (6).

More recently, 16S rRNA gene amplicon sequencing approaches have been applied to the analysis of samples from the middle ears of healthy adults and children. Neeff and colleagues assessed the healthy middle ears of 10 adults and 12 children undergoing either cochlear implantation (CI) or benign brain tumor resection (vestibular schwannoma) (9), reporting the detection of a variety of bacterial taxa, including the genera *Novosphingobium*, *Staphylococcus*, *Streptococcus*, *Escherichia*-*Shigella*, *Burkholderia*, *Propionibacterium*, and *Pseudomonas*. However, they were unable to isolate any of these taxa by culture. Minami and colleagues (10) employed 16S rRNA gene amplicon sequencing to assess middle ear mucosal swabs from 32 adults and 35 children. Again, they reported scant bacterial isolation by culture, but a diversity of bacterial taxa were detected by sequencing, particularly, members of the phylum *Proteobacteria* and the genus *Staphylococcus*.

The contrasting findings obtained with scanning electron microscopy (SEM), culture-based, and sequencing-based approaches raise the possibility that bacterial signals detected using the latter are artefactual. Our aim was to assess whether bacteria previously reported by amplicon sequencing in middle ear samples obtained under sterile conditions represented genuine bacterial populations or whether they were likely to represent analytical artifacts derived through the application of contaminated reagents to samples from a very-low-biomass context. To achieve this, we performed a comprehensive investigation of middle ear microbiology in the absence of disease in a manner not reliant on any single analytical approach. We have utilized a combination of culture-based diagnostic microbiology, microscopy, quantitative PCR, and a low-biomass 16S rRNA gene sequencing methodology.

## RESULTS

### Assessment of bacterial load.

**FIG 1:**
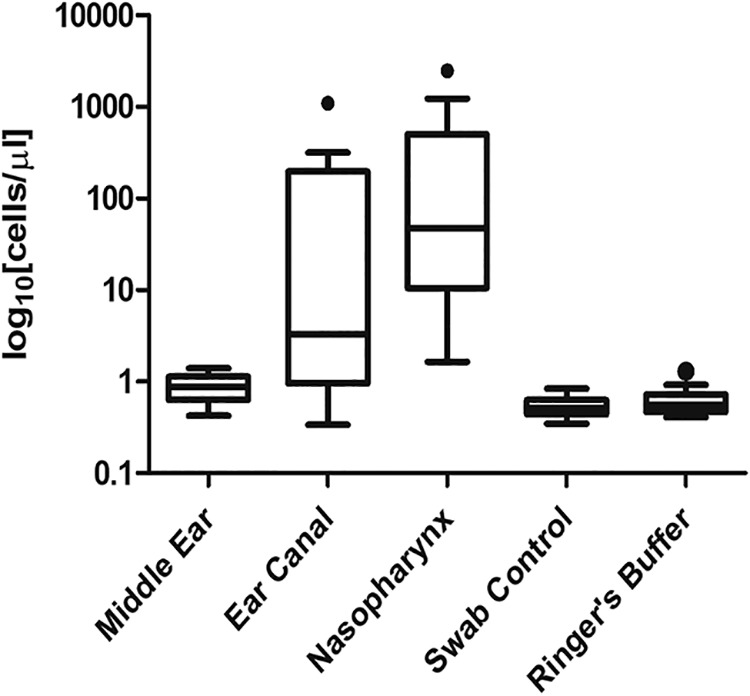
Tukey’s box plot showing sample bacterial loads, as determined by quantitative PCR. Middle ear samples had a median of 0.9 log10 cells/μl and interquartile range (IQR) of 0.6 to 1.1, ear canal samples had a median of 3.3 and IQR of 0.9 to 195.8, while nasopharyngeal samples had a median of 47.5 and IQR of 10.5 to 502.0. Swab controls had a median of 0.5 log10 cells/μl with an IQR of 0.4 to 0.6. Ringer’s buffer had a median of 0.6 log10 cells/μl with an IQR of 0.5 to 0.7.

Assessment was performed on middle ear (ME) swabs from 18 subjects (Table 1). Of these, matching nasopharyngeal (NP) and ear canal (EC) swabs were available for 17 and 16 subjects, respectively. Bacterial load was determined for all samples by quantitative PCR (qPCR) (Fig. 1). Levels of bacteria detected within EC and NP samples were consistent with our previous report (median of 30,304 copies/μl from EC, 20,479 copies/μl from NP) (11). In contrast, bacterial amplification from ME swabs was not significantly above background levels seen in extracts from negative controls (*P* > 0.05).

**TABLE 1:** Patient characteristics

Characteristic	Value
No. of patients	25
Age (yr) (mean [range])	61 (21–82)
Sex (*n*)	
Male	14
Female	11
Operation (*n*)	
Cochlear implant	21
Stapedotomy	3
Vestibular schwannoma resection	1
Indication for surgery (*n*)	
Sensorineural hearing loss	21
Otosclerosis	3
Vestibular schwannoma	1
Middle ear mucosa (*n*)	
Normal	22
Thickened	2
Inflamed	0
Granulomatous	0
Unknown	1
Temporal bone abnormality (*n*)	
No	21
Yes	4
Otosclerosis (*n*/total)	3/7
Previous temporal bone fracture	1/7

### Microbiota composition.

**FIG 2:**
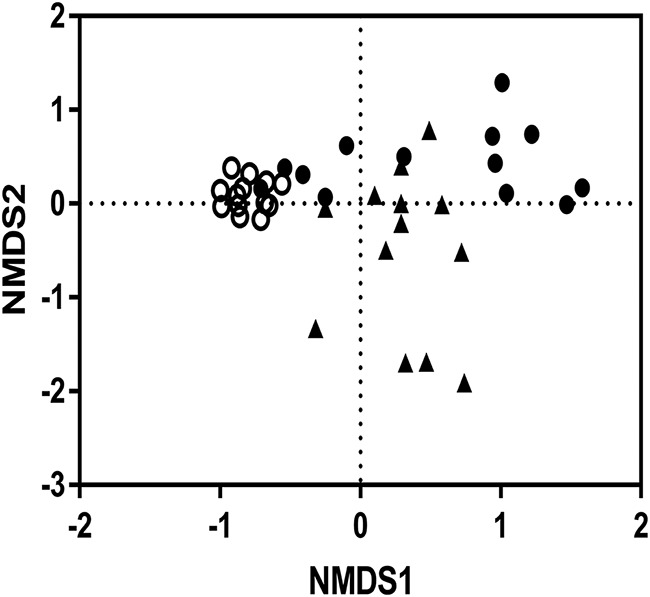
NMDS plot based on Bray Curtis dissimilarity differences for subjects where middle ear (○), ear canal (●), and nasopharyngeal samples (▲) were all available (*n* = 13).

16S rRNA gene amplicon sequencing was performed on DNA extracts where all three sample types were available for an individual subject (*n* = 16) and visualized by nonmetric multidimensional scaling (NMDS) (Fig. 2). Microbiota compositions differed significantly between each of the three sample types assessed [*P*(perm) = 0.0001, pseudo-F = 6.2549, 9,907 permutations]. In addition, significant differences in dispersion were observed between the three sampling sites [pseudo-F = 18.94, *P*(perm) = 0.001), including in pairwise comparisons of ME and NP samples [Student’s *t* = 5.74, *P*(perm) = 0.0001] and between ME and EC samples [*t* = 5.90, *P*(perm) = 0.0001].

*Corynebacterium* (range, 0.08% to 94.74%), *Staphylococcus* (0.08% to 94.74%), and *Propionibacterium* (0.08% to 94.74%) were predominant in ear canal samples while nasopharyngeal samples were dominated by *Haemophilus* (0.08% to 94.74%), *Streptococcus* (0.08% to 94.74%), and *Granulicatella* (0.08% to 94.74%). In contrast, the predominant bacterial genera in the middle ear samples were *Pseudomonas* (range, 0% to 37.31%) and *Methylobacterium* (range, 0% to 18.51%).

**FIG 3:**
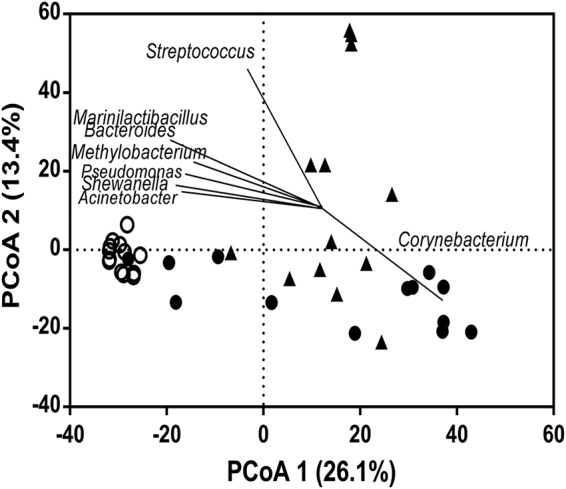
Canonical correspondence biplots for microbiota. ○, middle ear; ●, ear canal; ▲, nasopharynx. Biplot lines for bacterial taxa show the direction of increase, with the length of each line indicating the degree of correlation with ordination axes.

**FIG 4:**
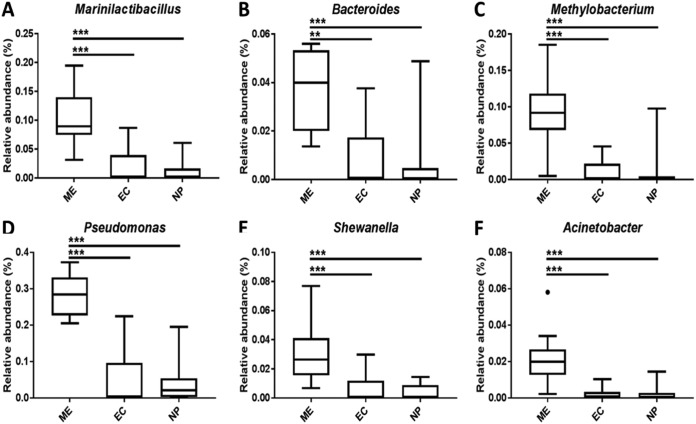
Tukey’s box plots showing relative abundances of taxa strongly associated with middle ear microbiota, as defined by 16S rRNA gene amplicon sequencing. ME, middle ear; EC, ear canal; NP, nasopharynx.

To assess the contribution of individual taxa to microbiota dispersion, the degree of correlation between taxa and ordination axes was visualized using a principal-coordinate analysis biplot (Fig. 3). Taxa whose distributions contributed substantially to the differences in microbiota dispersions between sample types included *Corynebacterium* (more prevalent in ME and NP), *Marinilactibacillus*, *Bacteroides*, *Methylobacterium*, *Pseudomonas*, *Shewanella*, and *Acinetobacter*. Of these, the relative abundances of taxa overrepresented in middle ear samples are shown in Fig. 4.

Sequencing reactions performed on negative controls yielded bacterial signals (see Fig. S4 in the supplemental material). While bacterial profiles from these controls were not identical to those derived from middle ear samples, they returned sequences corresponding to *Pseudomonas* and *Ruminococcaceae* (the latter being detected in three middle ear samples but not in ear canal or nasopharyngeal samples).

### Linear regression analysis of potential contaminant taxa.

The bacterial taxa that contributed substantially to middle ear microbiota profiles either were identified previously as common sequencing reagent contaminants (*Methylobacterium*, *Pseudomonas*, and Acinetobacter) or have little biological plausibility in this context (*Shewanella* and *Marinilactibacillus*) (12, 13). Such contaminants commonly display a negative relationship between their relative abundance and sample bacterial load, becoming relatively more prevalent where there is reduced bacterial template for PCR amplification. Therefore, a further analysis was performed to assess the relationship between *Pseudomonas*, the most prevalent potential contaminant taxa, and bacterial load (as determined by qPCR).

*Pseudomonas* operational taxonomic units (OTUs) displayed a significant negative linear relationship with square-root-transformed bacterial loads in middle ear samples (see Fig. S1A), strongly suggesting contamination. The absence of heteroscedasticity (equal variance) (studentized Breusch-Pagan [BP] test = 0.017619, df = 1, *P* = 0.8944) (Fig. S1B) and the normal nature of this relationship (Fig. S1C) support the appropriateness of the model. In contrast, while a similar negative linear relationship between *Pseudomonas* OTU abundance and transformed bacterial loads was displayed in nasopharyngeal samples (Fig. S1D), there was relatively strong heteroscedasticity (BP = 1.1277, df = 1, *P* value = 0.2883) (Fig. S1E) and a largely normal relationship (Fig. S1F). This distribution is consistent with the presence of both genuine pseudomonal populations in the nasopharynx and signal derived from contamination, a pattern that was replicated in samples from the ear canal (see Fig. S2A to C). In contrast to those for *Pseudomonas*, *Staphylococcus* OTUs in ear canal samples displayed a positive relationship between relative abundance and bacterial load, with a strong linear relationship (see Fig. S3A to C), strongly suggesting that these populations are genuine.

10.1128/mSphere.00456-19.1 FIG S1 Linear regression analysis indicating negative relationship between *Pseudomonas* taxon and bacterial loads as determined by 16S rRNA qPCR. (A) Linear plot of *Pseudomonas* taxon from middle ear. (B) Residual plot of *Pseudomonas* taxon from middle ear. (C) Quantile plot of *Pseudomonas* taxon from middle ear. (D) Linear plot of *Pseudomonas* taxon from nasopharynx. (E) Residual plot of *Pseudomonas* taxon from nasopharynx. (F) Quantile plot of *Pseudomonas* taxon from nasopharynx. Download FIG S1, TIF file, 14.8 MB. Copyright © 2019 Jervis-Bardy et al. 2019 Jervis-Bardy et al. This content is distributed under the terms of the Creative Commons Attribution 4.0 International license.

10.1128/mSphere.00456-19.2 FIG S2 Linear regression analysis of *Pseudomonas* taxon from ear canal, visualized through a linear plot (A), residual plot (B), and quantile plot (C). Download FIG S2, TIF file, 14.8 MB. Copyright © 2019 Jervis-Bardy et al. 2019 Jervis-Bardy et al. This content is distributed under the terms of the Creative Commons Attribution 4.0 International license.

10.1128/mSphere.00456-19.3 FIG S3 Linear regression analysis of *Staphylococcus* taxon from ear canal, visualized through a linear plot (A), residual plot (B), and quantile plot (C). Download FIG S3, TIF file, 14.8 MB. Copyright © 2019 Jervis-Bardy et al. 2019 Jervis-Bardy et al. This content is distributed under the terms of the Creative Commons Attribution 4.0 International license.

10.1128/mSphere.00456-19.4 FIG S4 Relative abundances of bacterial OTUs identified in DNA in reagent and negative-control extracts. Download FIG S4, TIF file, 14.8 MB. Copyright © 2019 Jervis-Bardy et al. 2019 Jervis-Bardy et al. This content is distributed under the terms of the Creative Commons Attribution 4.0 International license.

### Microscopy and bacterial culture.

The findings from our molecular analysis, including the distribution of detected taxa, their abundance, and their biological plausibility, strongly suggest the absence of any substantial bacterial populations in the healthy middle ear. Instead, these findings are consistent with contamination of sequencing reactions, as reported widely in other low-biomass contexts. To further investigate whether any evidence of bacteria within the healthy middle ear could be obtained, two additional analytical approaches were applied to samples obtained from 7 subjects: Gram stain and microscopic visualization, and standard diagnostic bacterial culture. Bacteria were detected in ear canal or nasopharyngeal samples from all seven individuals using at least one approach (Table 2). In contrast, there was no detection of bacteria in any middle ear sample, either by microscopy or culture.

**TABLE 2:** Detection of bacteria by microscopy and culture

Patient	Gram stain and microscopy^*a*^			Culture^*b*^		
	ME	EC	NP	ME	EC	NP
1	−	+	++	−	++	+++
2		+	−		++	+
3	−	−	−	−	++	+++
4	−	−	+	−	−	+
5	−	+	+	−	++	++
6	−	++	++	−	++	++
7	−	−	+	−	−	+
						
No. positive/total	0/6	4/7	5/7	0/6	5/7	7/7

a ME, middle ear; EC, ear canal; NP, nasopharynx; −, no bacteria seen; +, one bacterium per 100× field; ++, 2 to 30 bacteria per 100× field.

b −, no growth; +, scant growth; ++, moderate growth; +++, heavy growth.

## DISCUSSION

Our investigation aimed to assess whether bacteria previously reported by amplicon sequencing in middle ear samples obtained under sterile conditions represented genuine bacterial populations or whether they were likely to represent analytical artifacts derived through the application of contaminated reagents to samples from a very-low-biomass context. Previous reports of healthy middle ear microbiome are notable because many of the reported taxa were not detected by parallel culture-based analysis, despite being species that are readily culturable using standard approaches (9, 10). Furthermore, many of the reported taxa are genera that are commonly associated with contamination of sequencing reagents and which are very unlikely to be present within the human body (e.g., *Bradyrhizobium*). To address this, we combined a range of approaches, including quantitative PCR, amplicon sequencing, dispersion analysis, microscopy, and diagnostic culture, to directly assess the evidence that bacterial signal detected in the healthy middle ear using molecular approaches is artefactual.

Bacterial quantification based on qPCR indicated that bacterial abundance in the middle ear was significantly lower than in the ear canal or nasopharynx and was not higher than negative controls (DNA extracts from unused swabs and sterile irrigation buffer).

Microbiota profiles from middle ear samples were significantly different in composition from either ear canal or nasopharyngeal samples. 16S rRNA gene amplicon sequencing profiles from nasopharyngeal and ear canal samples were consistent with previous microbiological assessments of these contexts (14, 15). For example, bacteria detected in the ear canal were predominately those associated with the skin, including members of the *Corynebacterium*, *Staphylococcus*, and *Propionibacterium* genera. Nasopharyngeal samples were dominated by *Haemophilus*, *Streptococcus*, and *Granulicatella* genera. In contrast, microbiota profiles from middle ear samples were dominated by taxa that either were identified previously as sequencing agent contaminants (*Methylobacterium*, *Pseudomonas*, and *Acinetobacter*) or have little biological plausibility in this context, such as genera most commonly associated with extreme marine environments (*Shewanella*, *Marinilactibacillus*, and *Psychrobacter*) (12, 13). The likely spurious nature of bacterial signal in middle ear samples was further supported by the detection of *Pseudomonas* and *Ruminococcaceae* OTUs in negative controls.

Spurious OTUs, arising through contamination, can be distinguished from genuine bacteria by their relative abundance in relation to the sample biomass. As the amount of DNA in sequencing reaction falls, rarer community members cease to be detected, with the relative abundance of dominant taxa increasing as a natural consequence. However, signal derived from reagent contamination, which should remain a constant feature of all sequencing reactions regardless of genuine template concentration, behaves differently. The relative abundance of these OTUs tends to be low in high-biomass samples, where it represents only a small portion of total template (11). As sample biomass falls, the relative contribution of this spurious signal increases. Assessment of the relationship between *Pseudomonas* and bacterial load within the middle ear samples showed just such a relationship. In contrast, *Staphylococcus*, which was isolated from the ear canal of a number of subjects, behaved in a manner consistent with its genuine presence within EC samples.

In addition to bacterial DNA concentrations that were no higher than negative controls, and microbiota composition aligning with taxa shown previously to be commonly spurious, microscopy failed to detect any bacterial cells in middle ear samples. This was our finding despite the use of culture conditions appropriate for a majority of detected taxa and positive cultures resulting from ear canal and nasopharyngeal swabs.

This study had limitations that should be considered. First, while the burden of middle ear disease is greatest in childhood, our study was performed on samples from an adult cohort. Our broad aim was to examine the evidence of bacteria in the healthy middle ear of subjects irrespective of age. We cannot say, based on our analysis, whether findings would be identical in a pediatric cohort range. However, identifying important methodological considerations for the characterization of middle ear samples in general is an essential step toward this. Second, while we can conclude there was no evidence of viable bacterial cells using our techniques, whether bacteria might be identified using more advanced technologies is not known.

Our findings cast substantial doubt on previous reports of a healthy middle ear microbiome using 16S amplicon sequencing, which reported taxa in almost all instances that have been shown to be common sequencing artifacts in other contexts. Our findings further underline the importance of careful consideration of amplicon sequence data derived from very-low-biomass contexts.

## MATERIALS AND METHODS

### Sample collection.

Ethical approval for the study was obtained from the Southern Adelaide Clinical Human Research Ethics Committee (approval number 463.15; HREC/15/SAC/452). Intraoperative swabs were collected from the middle ear (ME), nasopharynx (NP), and external ear canal (EC) regions of 25 patients undergoing surgical procedures where access to a noncontaminated healthy middle ear was established (see Table 1 for patient characteristics).

The majority of swabs were collected from patients undergoing cochlear implantation. Swabs were also obtained from patients undergoing a stapedotomy procedure and during mastoidectomy as part of a translabyrinthine vestibular schwannoma resection.

Intraoperative NP and EC swabs were collected in a uniform manner for all procedures. Following general anesthesia, NP samples were collected by passing a sterile pediatric FLOQSwab (Copan, Murrieta, CA, USA) along the floor of the nasal cavity into the nasopharynx, and then keeping it *in situ* for 5 s while rotating 180°. FLOQSwabs were also used for EC specimens by passing the swabs through the external meatus under direct vision to the deep ear canal skin and rotating 180° for 5 s without making contact with the tympanic membrane. For procedures involving a cortical mastoidectomy (cochlear implant and translabyrinthine vestibular schwannoma resection), ME swabs were obtained after completing cortical mastoidectomy by passing a FLOQSwab into the mucosa of the attic region of the middle ear upon visualization of the ossicular chain. For the ME swab obtained during the stapedotomy procedure (a surgical approach to the middle ear through the ear canal), a FLOQSwab was passed through the ear canal under microscopic vision into the mucosa of the mesotympanic region of the middle ear. Care was taken under direct microscopic vision to avoid contamination with the external ear canal when placing the swab in the middle ear during the transcanal approach. All samples were stored on ice and transferred to −80°C at the completion of the surgical procedure.

### Diagnostic culture, Gram staining, and microscopy.

Swabs were transported immediately to the on-site laboratory for processing. The first swab was used to prepare a Gram stain slide, which was examined for bacteria at ×100 magnification under an oil-immersion objective lens. A second swab was cultured under conditions to allow for growth of fastidious organisms, anaerobes, and aerobic Gram-negative organisms. Specifically, swabs were plated and incubated on horse blood agar (HBA; bioMérieux, Australia) under anaerobic conditions (10% H_2_, 10% CO_2_, 80% N_2_) at 37°C, on chocolate agar (PolyViteX; bioMérieux, Australia) in an enriched carbon dioxide atmosphere (5% CO_2_) at 37°C, and on MacConkey agar (MAC; bioMérieux, Australia) in ambient air at 37°C. Plates were examined for bacterial growth after 24 h and 48 h. If growth was noted, colonies were identified by using matrix-assisted laser desorption ionization–time of flight mass spectrometry (Bruker Daltonik MALDI Biotyper; Bruker Biosciences Pty Ltd., Preston, VIC, Australia).

### DNA extraction, quantitative PCR, and 16S rRNA gene amplicon sequencing.

Total DNA was extracted from all clinical samples using a methodology designed for low-biomass contexts (11). Unused swabs and unused Ringer’s solution, used in the irrigation of the middle ear, were used as negative controls and were extracted in parallel with clinical specimens. In brief, a QIAamp kit (Qiagen, Chadstone, VIC, Australia) was used in conjunction with enzymatic lysis and physical disruption. Total DNA was eluted in 100 μl of sterile water and quantified fluorometrically with a Qubit dsDNA HS assay kit (Life Technologies, Melbourne, Australia).

Total bacterial load from samples were assessed using qPCR assay targeting the 16S rRNA gene as described previously (16). The qPCR conditions comprised 50°C for 2 min and 95°C for 10 min, followed by 40 cycles of 95°C for 15 s and 60°C for 1 min. Melt curve analysis was then performed under the following conditions: 95°C for 15 s, followed by an initial stage temperature of 60°C for 1 min and a final temperature of 95°C for 15 s, with readings recorded at increments of 0.05°C/s. Standard curves were generated for each qPCR reaction based on serial dilutions of Escherichia coli genomic DNA for the 16S rRNA gene.

16S rRNA gene sequencing was performed using the Illumina MiSeq platform (Illumina, Scoresby, VIC, Australia). Libraries were generated by amplifying the V1-V3 hypervariable region of the 16S rRNA gene using fusion degenerate primers 27F (5′-TCGTCGGCAGCGTCAGATGTGTATAAGAGACAGAGRGTTTGATCMTGGCTCAG-3′) and 519R (5′-GTCTCGTGGGCTCGGAGATGTGTATAAGAGACAGGTNTTACNGCGGCKGCTG-3′) with ligated overhang Illumina adapter consensus sequences. Library generation was performed according to the Illumina 16S Metagenomic sequencing library preparation guide with minor modifications, as described previously (17). A MiSeq V3 reagent kit (Illumina, Scoresby, VIC, Australia) was used for library sequencing. Reads from Illumina sequencing were used as raw data for bioinformatic analyses. Mock bacterial community controls, samples of surgical irrigation solution, and blank swabs were including as controls.

### Biostatistical analysis.

Mean relative abundances of top OTUs were calculated for the ME, NP, and EC swabs. OTU-level analyses were performed on OTUs with mean relative abundances in the top 20 of each sample type, provided the cutoff did not exclude OTUs with greater than 1.5% mean relative abundance in a sample type. A nonparametric Kruskal-Wallis test was used to determine the significance of variation in diversity across sample types. Bray-Curtis (BC) similarity matrices were generated, and nonmetric multidimensional scaling was employed for cluster analysis. A permutational multivariate analysis of variance (PERMANOVA) test was performed in PRIMER to test whether there was a statistically significant difference between the bacterial communities in the ME, EC, and NP samples (with sample types as a fixed factor).

Bacterial taxa that were identified as potential contaminants were further assessed by linear regression. Square-root-normalized taxon relative abundance was plotted against bacterial load Z-score. The linear regression was further assessed for heteroscedasticity and normality using the lmtest package (version 0.9-36) in R. For species where outliers were present, analyses were repeated with outliers excluded to confirm that the findings were not substantially altered.

### Data availability.

Sequence data were submitted to the Sequence Read Archive under accession number PRJNA523067.
