# Supplementary figures and images for: Examining the Evidence for an Adult Healthy Middle Ear Microbiome
Source: mSphere. 2019 Sep 4;4(5):e00456-19. doi: 10.1128/mSphere.00456-19 (PMC6731531; doi:10.1128/mSphere.00456-19)

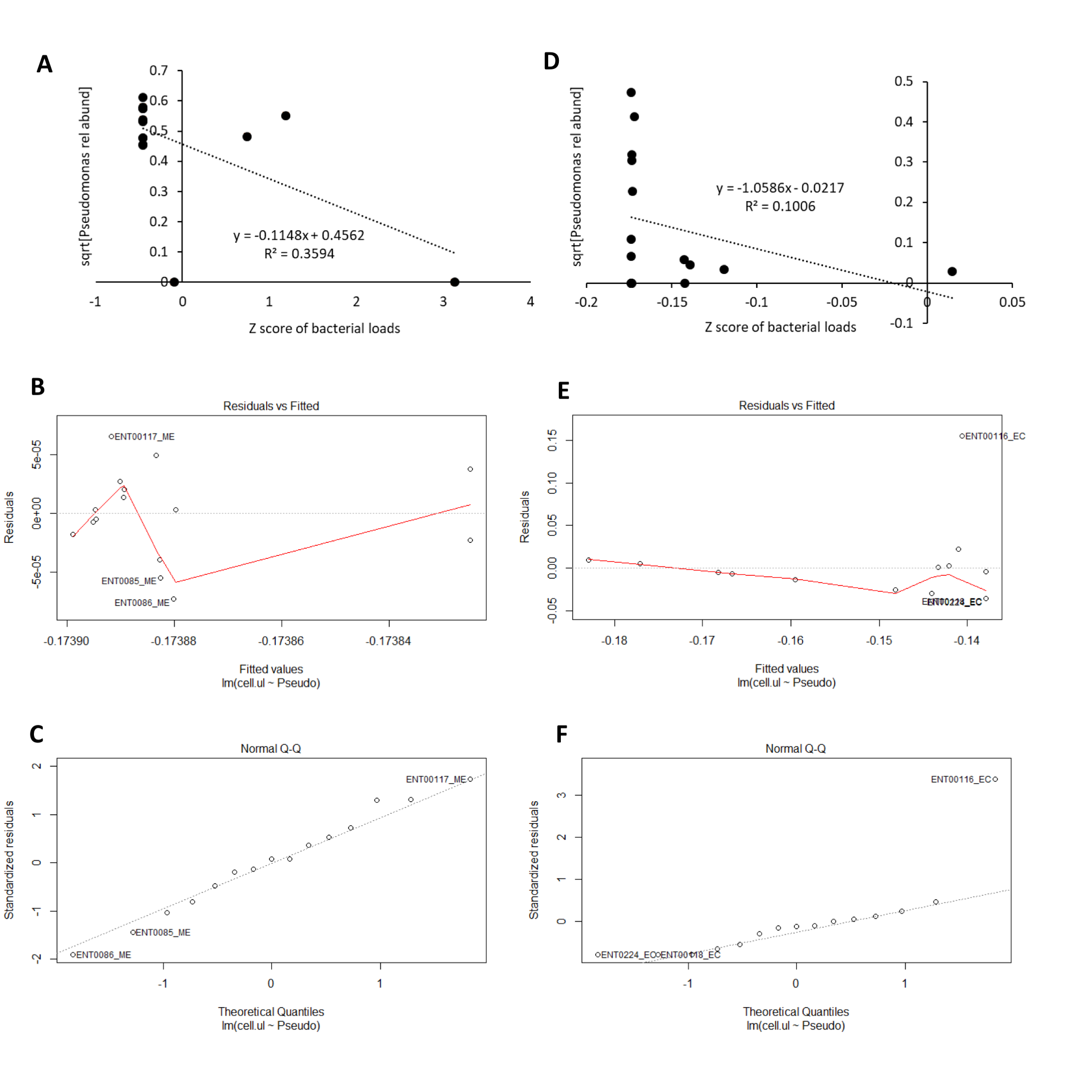

Supplement: FIG S1 [file mSphere.00456-19-sf001.tif]

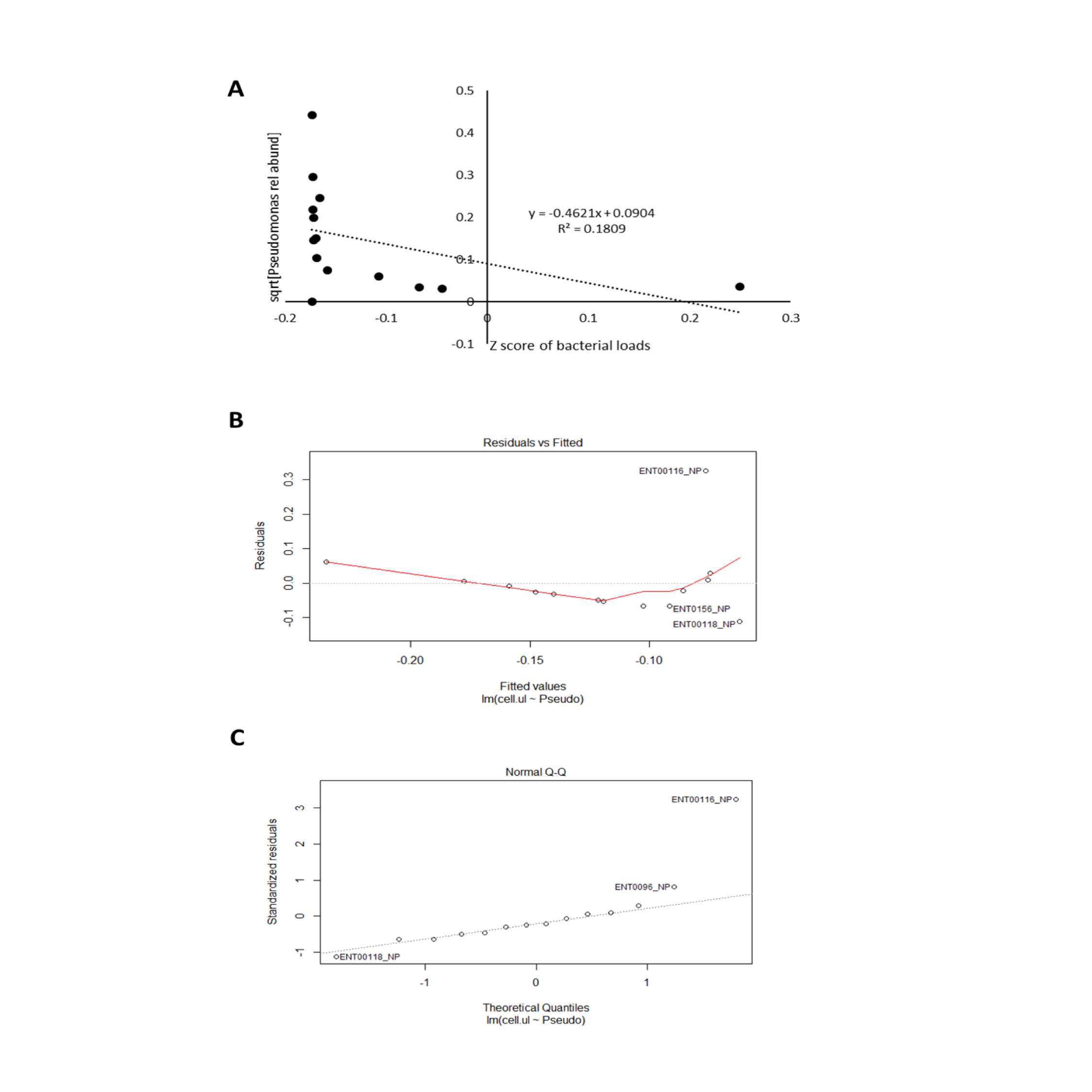

Supplement: FIG S2 [file mSphere.00456-19-sf002.tif]

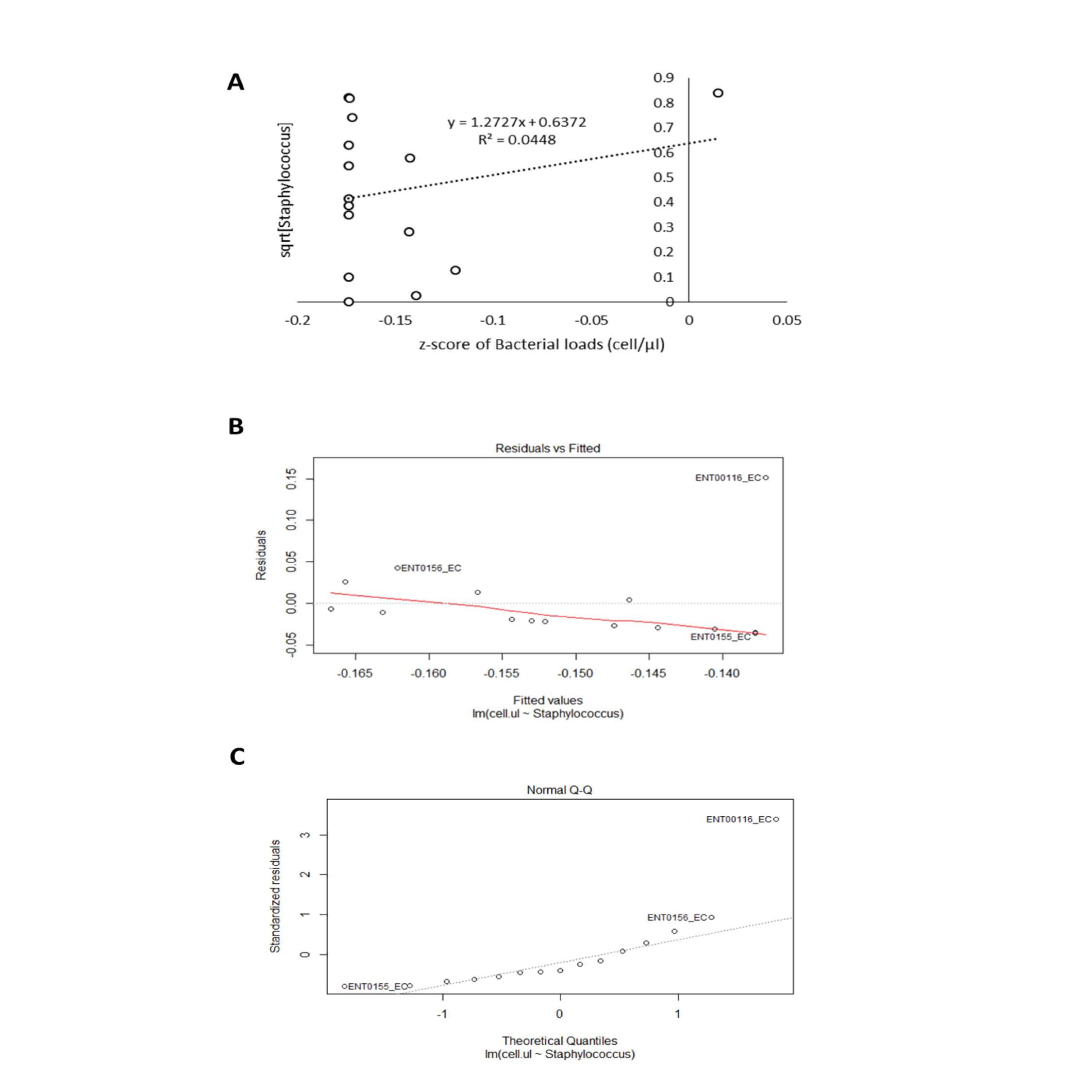

Supplement: FIG S3 [file mSphere.00456-19-sf003.tif]

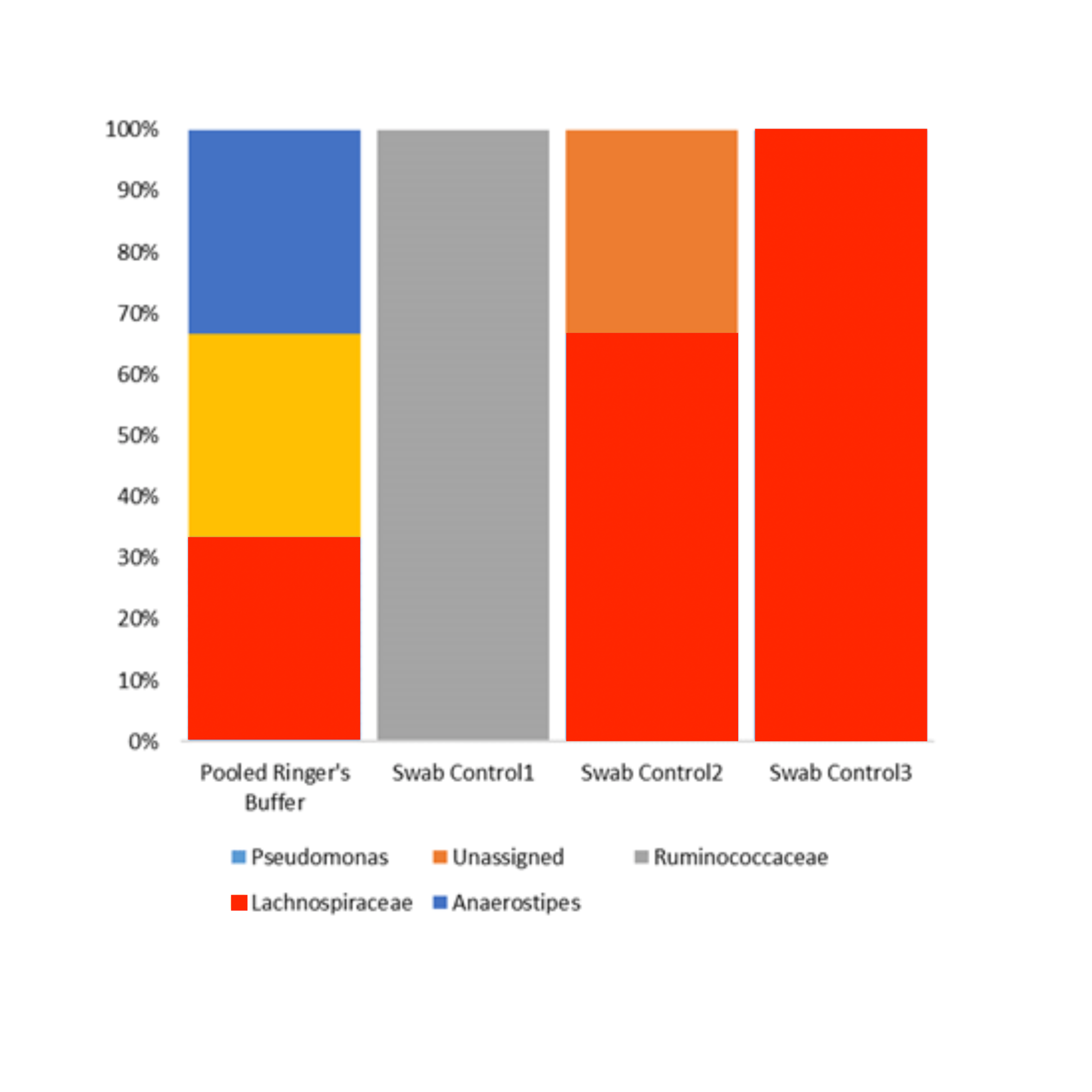

Supplement: FIG S4 [file mSphere.00456-19-sf004.tif]
